# Trephine Stoma in High-Risk Patients: Feasibility and Short-Term Outcomes

**DOI:** 10.3390/jcm15187148

**Published:** 2026-09-15

**Authors:** Serdar Gumus, Ibrahim Cogal, Halil Gunes, Nurefsan Sadıkoglu, Ishak Aydın, Ismail Cem Eray

**Affiliations:** 1 Department of Surgical Oncology, Faculty of Medicine, Cukurova University, Adana 01330, Turkey; 2 Department of General Surgery, Faculty of Medicine, Cukurova University, Adana 01330, Turkey; 3 Department of Anesthesiology and Reanimation, Faculty of Medicine, Cukurova University, Adana 01330, Turkey

**Keywords:** geriatric surgery, trephine stoma, frailty, bowel obstruction, minimally invasive technique, stoma

## Abstract

**Background/Objectives:** In older patients undergoing abdominal surgery, perioperative risk is influenced not only by chronological age but also by frailty, comorbidity burden, and diminished physiological reserve. These factors may limit tolerance of conventional abdominal surgery and increase interest in less invasive surgical approaches in high-risk patients. The trephine approach provides limited surgical access without routine laparotomy, but evidence regarding its use in this population remains limited. This study evaluated the technical feasibility and short-term outcomes of the trephine approach in high-risk older patients. **Methods:** Patients aged ≥65 years who underwent stoma surgery using the trephine approach at a tertiary referral center between January 2016 and January 2026 were retrospectively reviewed. During the study period, 488 patients underwent stoma surgery; of these, 10 (2.0%) were managed using the trephine approach and formed the study cohort. We used preoperative CT for surgical planning in all cases, with intraoperative endoluminal guidance when required. We analyzed demographic, perioperative, and short-term outcome data descriptively. **Results:** The mean age was 77.9 ± 9.6 years. Median ASA physical status was 4, median Clinical Frailty Scale score was 5.5, and median Charlson Comorbidity Index was 5.5. Eight patients (80%) underwent emergency surgery. The planned procedure was completed without conversion to laparotomy in 9/10 patients (90%), and bowel resection was performed through the same incision in 4/10 (40%). Median operative time was 32.5 min, and median hospital stay was 5 days. Major complications occurred in 3/10 patients (30%), and 30-day mortality was 2/10 (20%). **Conclusions:** In this small retrospective series of high-risk older patients, the trephine approach was technically achievable in most cases when used with preoperative CT-based planning and, when needed, intraoperative endoluminal guidance. These findings provide preliminary descriptive evidence regarding the feasibility of this approach in a highly selected clinical setting. Given the small sample size, lack of a comparator, and observed postoperative morbidity and mortality, no conclusions can be drawn about safety, comparative effectiveness, or patient-selection criteria.

## 1. Introduction

The growing older population worldwide is increasing the incidence of gastrointestinal conditions requiring surgical treatment [1]. Colorectal malignancies, sigmoid volvulus, diverticulitis, and various obstructive conditions have made stoma formation an increasingly common clinical need in older patients [2].

In this population, perioperative risk is determined not only by chronological age but also by frailty, comorbidity burden, sarcopenia, and diminished cardiopulmonary and physiological reserve [3]. These factors are associated with an increased risk of postoperative complications, prolonged hospital stay, and mortality after major abdominal surgery [4,5]. Consequently, less invasive surgical approaches may be considered in high-risk older patients with limited physiological reserve who may have reduced tolerance of standard abdominal surgery.

The trephine stoma was first described by Senapati and colleagues as a minimally invasive stoma technique that does not require laparotomy [6], and subsequent studies reported potential advantages such as shorter operative time and lower surgical trauma [7]. It has also been shown that resection and end colostomy can be performed via the trephine approach in debilitated, high-risk sigmoid volvulus cases [8]. Subsequent small series have supported the technical feasibility of the trephine approach [9,10], while highlighting limitations related to restricted visualization, difficulty in identifying and orienting the intended bowel segment, and the potential need for conversion to laparotomy [11,12,13,14]. Although laparoscopy-assisted [12] and endoscopy-assisted [15,16] modifications have been developed to mitigate these issues, the existing literature remains largely limited to small, retrospective, and heterogeneous patient series [17].

In older patients at high surgical risk, chronological age alone may provide insufficient information for operative decision-making. Contemporary geriatric surgical assessment increasingly recognizes frailty and physiological reserve as important determinants of perioperative risk [4,18,19]. Nevertheless, evidence regarding the use of the trephine approach specifically in high-risk older patients remains limited, particularly in the context of integrating physiological risk assessment with preoperative anatomical planning.

Therefore, this study aimed to describe the technical feasibility and short-term outcomes of the trephine approach in a small retrospective series of high-risk older patients. Particular attention was given to preoperative CT-based planning, the use of intraoperative endoluminal guidance, and completion of the planned procedure without conversion to laparotomy. We also described bowel resection through the same incision as a technical observation in selected cases.

## 2. Materials and Methods

### 2.1. Study Design and Patient Identification

This study was designed as a single-center, retrospective, observational case series. Adult patients who underwent stoma surgery at a tertiary referral center between January 2016 and January 2026 were retrospectively identified through the institutional database. During the study period, a total of 488 adult patients underwent stoma surgery. Among these patients, the 10 patients who underwent stoma surgery using the trephine approach constituted the case series.

In our clinical practice, the trephine approach was primarily considered for patients who were deemed to be at high surgical risk or to have limited capacity to tolerate the physiological burden of conventional abdominal surgery. Surgical risk was determined through a joint assessment by the surgical and anesthesiology teams, taking into account the patient’s overall clinical condition, physiological reserve, frailty, comorbidity burden, and ability to tolerate conventional abdominal surgery. No predefined numerical cutoff values for the American Society of Anesthesiologists (ASA) physical status classification or the Clinical Frailty Scale (CFS) were used to determine eligibility for the trephine approach.

**Figure 1:**
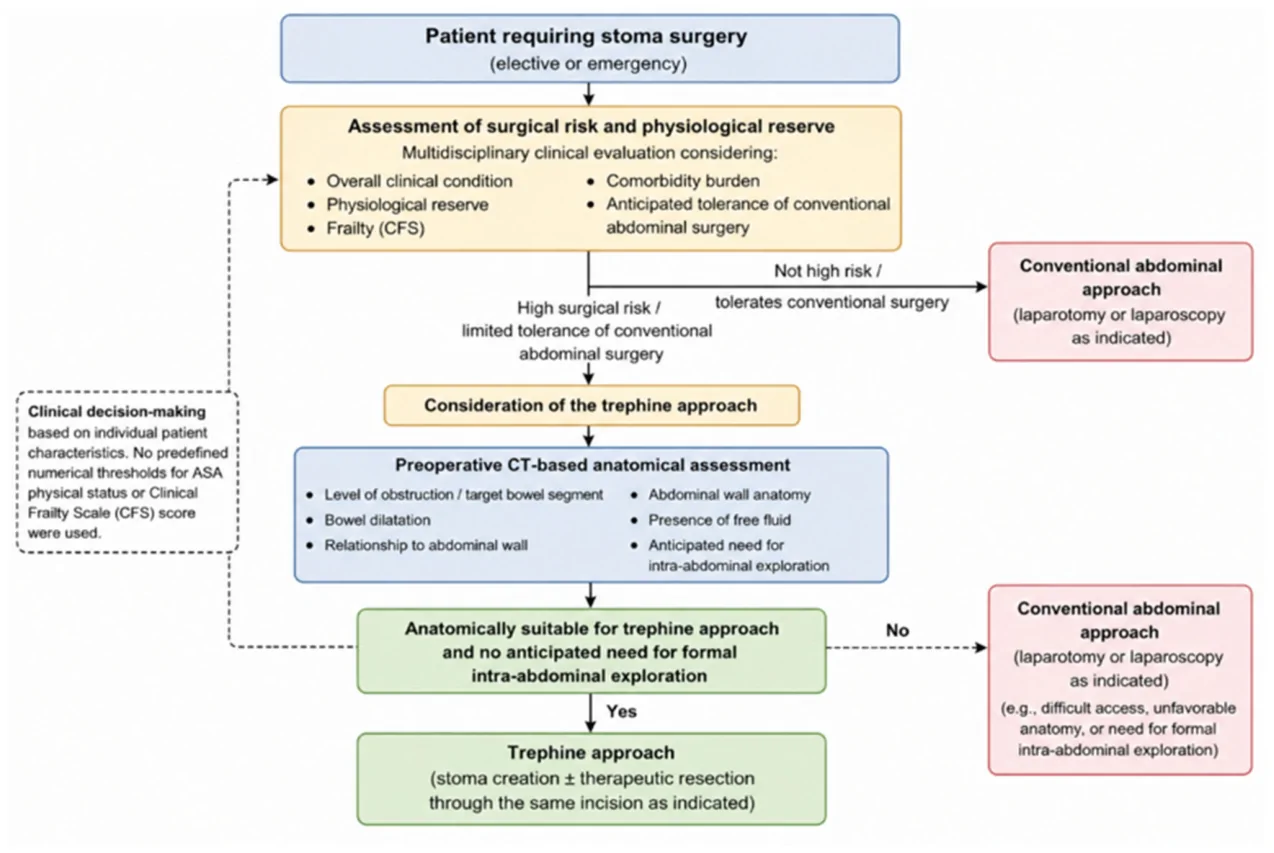
Clinical considerations for the use of the trephine approach in high-risk patients requiring stoma surgery. The decision to consider the trephine approach was based on individualized assessment of surgical risk and physiological reserve together with CT-based anatomical suitability.

In patients for whom the trephine approach was considered because of high surgical risk, the technical feasibility of the approach was assessed using preoperative computed tomography (CT). This assessment considered the level of obstruction and the target bowel segment, bowel dilatation, the relationship between the target bowel segment and the abdominal wall, abdominal wall anatomy, the presence of intra-abdominal free fluid, and the anticipated need for formal intra-abdominal exploration. The trephine approach was performed in patients who were considered anatomically suitable and in whom formal intra-abdominal exploration was not anticipated to be necessary. In patients with unsuitable anatomical conditions or in whom formal intra-abdominal exploration was anticipated to be necessary, a conventional abdominal approach was preferred according to clinical requirements. This clinical decision-making process is illustrated in Figure 1.

The Charlson Comorbidity Index was retrospectively calculated from the medical records to characterize the comorbidity burden and was not used in the original clinical decision-making process regarding the trephine approach. The fundamental patient-selection principles and operative approach remained consistent throughout the study period.

This study was prepared and reported in accordance with the PROCESS (Preferred Reporting of Case Series in Surgery) guideline for reporting surgical case series [20].

### 2.2. Preoperative Assessment and Surgical Planning

All patients underwent routine preoperative evaluation, including physical examination, laboratory investigations, anesthesiology assessment, and radiological imaging. The American Society of Anesthesiologists (ASA) physical status classification and the Clinical Frailty Scale (CFS) were routinely recorded preoperatively. The CFS was assessed jointly by the surgical and anesthesiology teams based on the patient’s clinical and functional status at the time of preoperative evaluation.

For surgical planning, all patients underwent abdominal computed tomography (CT). Intravenous contrast was used in patients with adequate renal function. In patients without complete obstruction, oral contrast was administered when necessary to better define the level of obstruction and the stenotic segment; however, oral contrast was not administered to patients with complete ileus because of the risk of aspiration.

**Figure 2:**
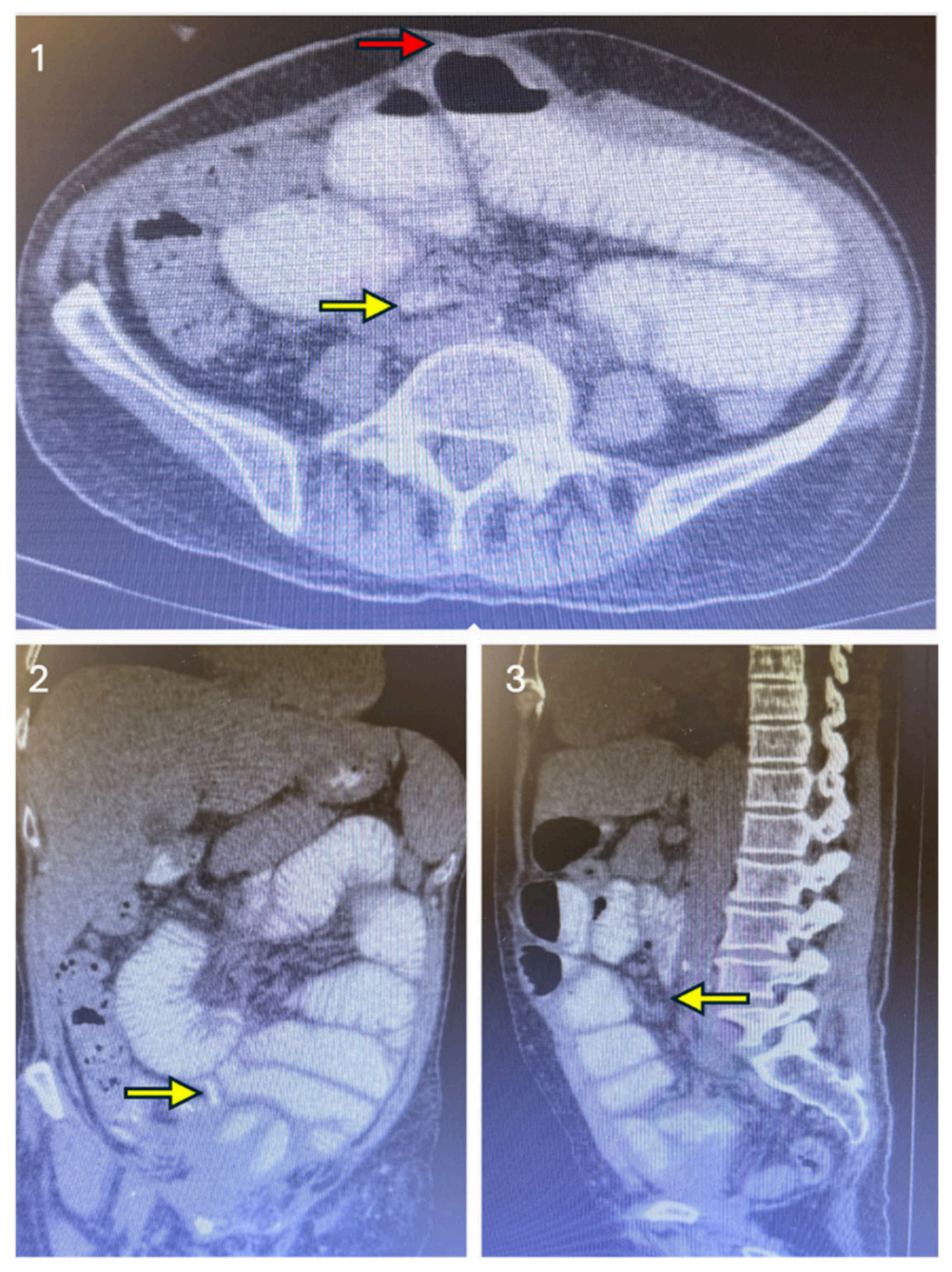
Preoperative computed tomography in a patient with a prior cystectomy for bladder tumor (Patient 10). (**1**) Axial slice: the red arrow indicates the planned trephine/stoma site, and the yellow arrow indicates the level of obstruction. (**2**) Coronal reformatted image: yellow arrow indicates the level of obstruction. (**3**) Sagittal reformatted image: yellow arrow indicates the level of obstruction. The obstructed segment was located approximately 30 cm proximal to the planned target segment and, based on CT measurement, approximately 200 cm distal to the ligament of Treitz; this localization supported the decision for loop ileostomy.

The surgeon and stoma care nurse marked the planned stoma site preoperatively. In determining the stoma site, the patient’s body habitus, previous abdominal incisions, mobility, sitting and standing positions, and postoperative stoma-care requirements were taken into consideration. A representative example of CT-based surgical planning is shown in Figure 2.

### 2.3. Surgical Technique

**Figure 3:**
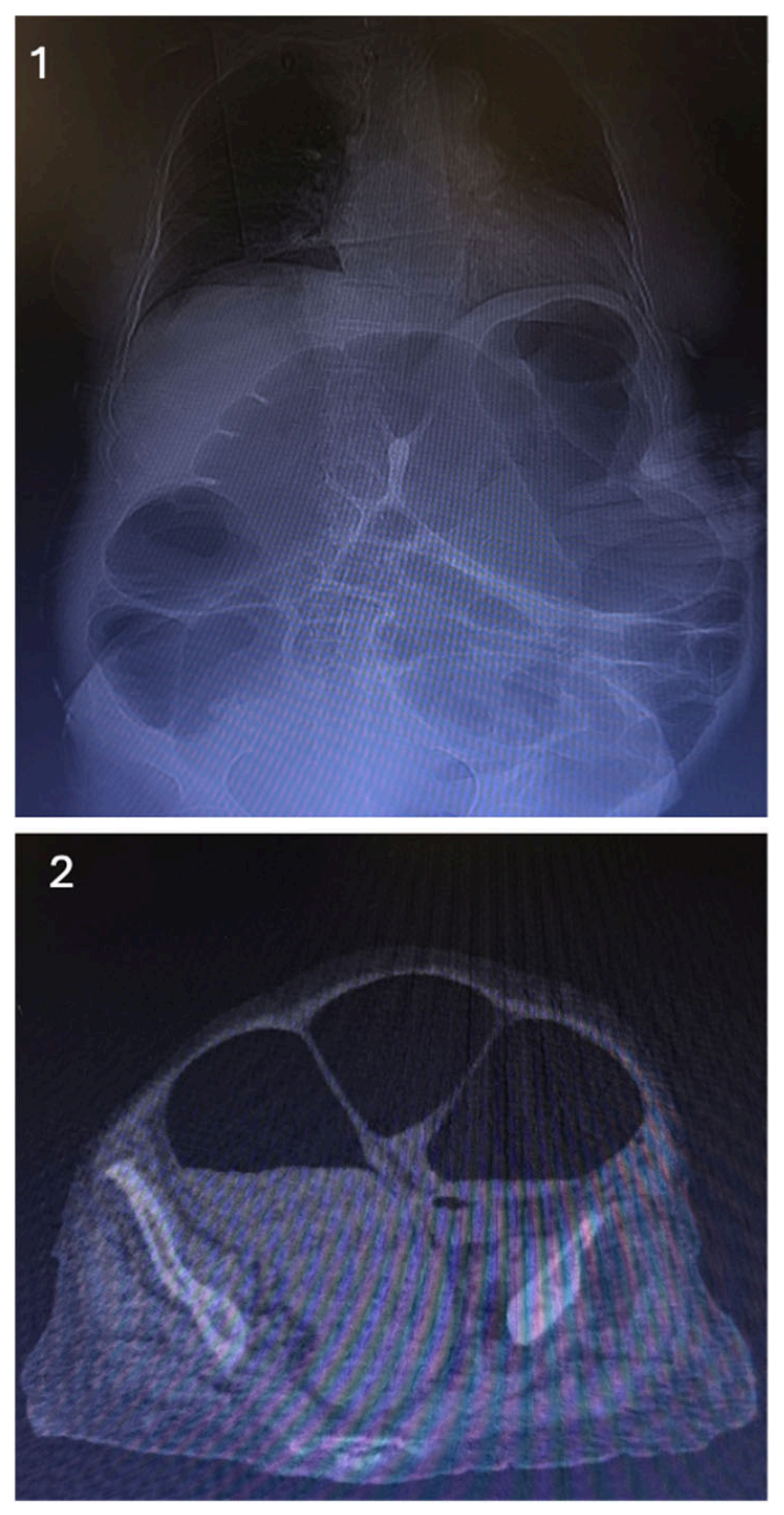
Preoperative imaging of a patient diagnosed with sigmoid volvulus. (**1**) Erect plain abdominal radiograph: a massively dilated, air-filled sigmoid colon segment, consistent with the classic “coffee bean” appearance of sigmoid volvulus. (**2**) Axial computed tomography slice: the same markedly dilated bowel segment and thinned wall structure are seen. These imaging findings directly correspond to the intraoperative exteriorization view shown in Figure 5.

**Figure 4:**
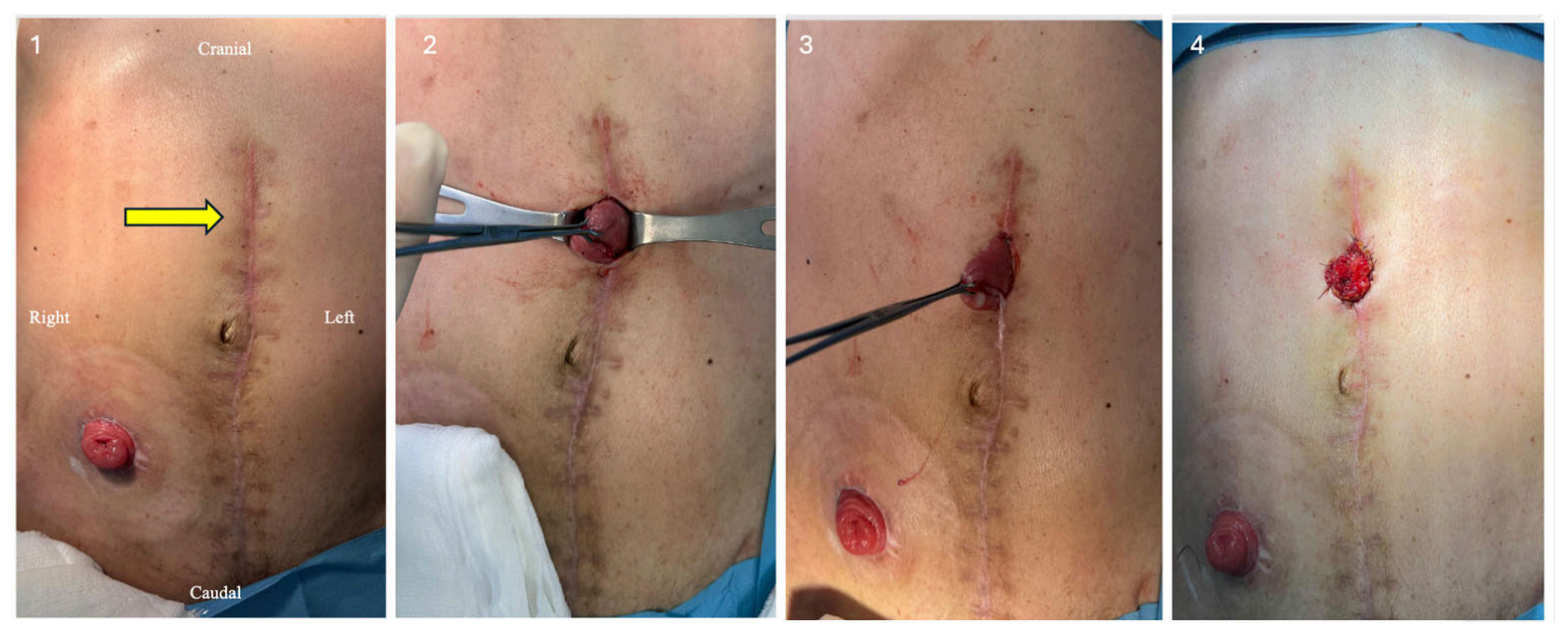
Trephine stoma created for new obstruction in a patient with a prior cystectomy and ileal conduit for bladder tumor. (**1**) Marking of the planned trephine site (yellow arrow); the previous midline scar and the existing ileal conduit stoma (lower left) are visible. (**2**) Exteriorization of the target bowel segment through the trephine opening using forceps. (**3**) Fixation of the segment at skin level. (**4**) Final appearance—the newly created trephine stoma alongside the pre-existing ileal conduit stoma.

**Figure 5:**
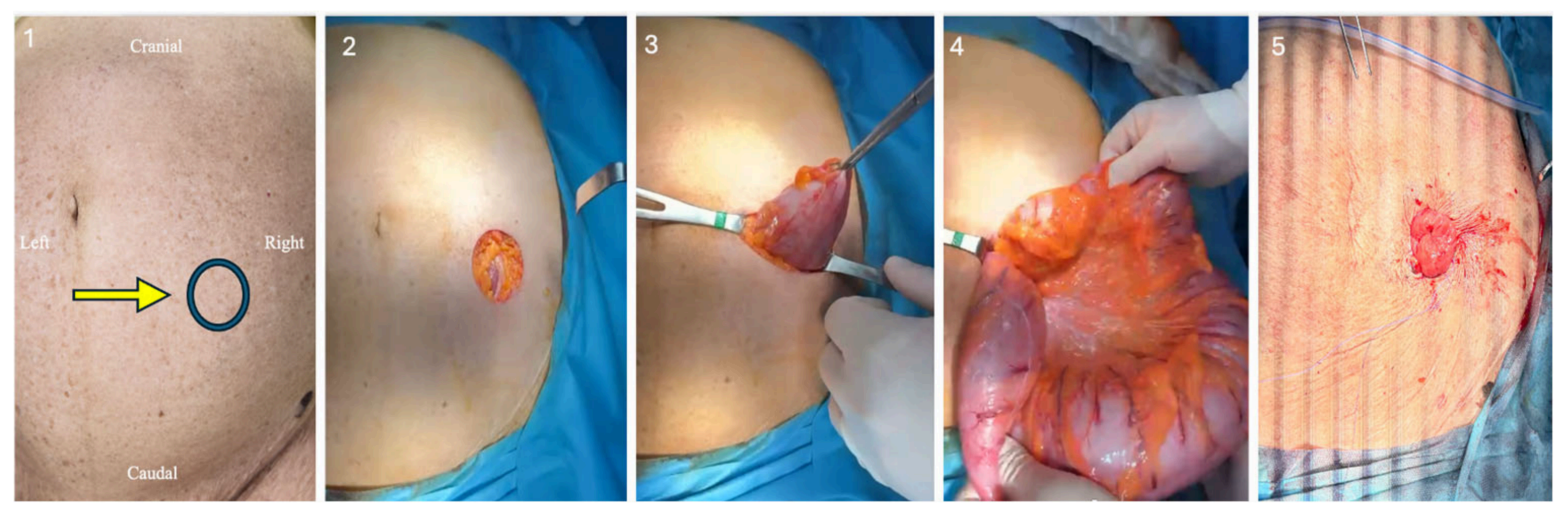
Intraoperative view of one of the patients who underwent resection through the same trephine opening for sigmoid volvulus (see Figure 3 for the preoperative imaging of the same patient). (**1**) The planned trephine site is outlined by the blue circle and indicated by the yellow arrow. (**2**) Opening after the trephine incision. (**3**) Exteriorization of the dilated, congested bowel segment using forceps. (**4**) Complete exteriorization of the markedly distended and redundant sigmoid colon segment—the pronounced mesenteric enlargement is notable; this view concretely illustrates the difficulty of laparoscopic manipulation and the limitation in trocar placement discussed in the Section 4. (**5**) Final appearance after resection and stoma maturation.

**Figure 6:**
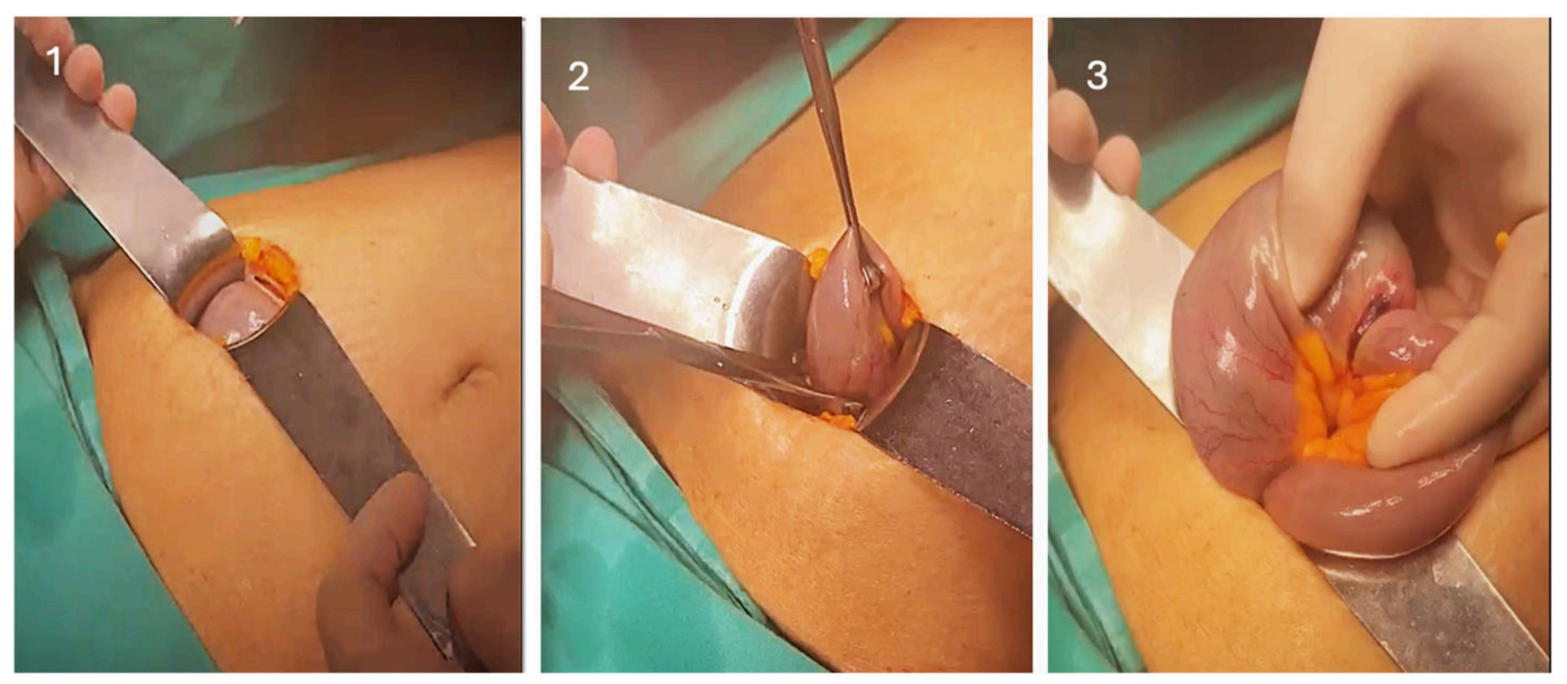
Intraoperative view of the patient (Patient 3) who underwent emergency surgery for obstruction due to an ileal tumor, who was intubated and in critical overall condition preoperatively. (**1**) Exposure of the trephine opening with retractors and visualization of the target small bowel segment. (**2**) Dissection of the segment and mesentery using forceps. (**3**) Complete exteriorization of the dilated small bowel segment and its mesentery.

In the trephine approach, patient positioning was determined according to the planned stoma site. For sigmoid colostomies, patients were routinely placed in the lithotomy position to allow transanal endoluminal assessment when necessary. A limited incision was made at the preoperatively marked stoma site, and the abdominal cavity was entered. The target bowel segment was then delivered through the incision. Representative preoperative imaging of sigmoid volvulus is shown in Figure 3. Figure 4 illustrates trephine stoma creation in a patient with a prior cystectomy and ileal conduit, Figure 5 presents the corresponding intraoperative sequence for the sigmoid volvulus patient shown in Figure 3, and Figure 6 illustrates the procedure in a critically ill patient with an ileal tumor.

After exteriorization of the colon, intraoperative endoluminal confirmation was used when the proximal and distal limbs could not be reliably distinguished. For this purpose, the distal colonic segment was identified and bowel orientation was confirmed by transanal rectoscopy, colonoscopy, or air insufflation. These methods were not routinely performed in all patients; rather, they were used as adjunctive intraoperative techniques when confirmation of bowel orientation was required, particularly in patients undergoing sigmoid colostomy.

In the four patients requiring bowel resection, we planned resection preoperatively based on CT findings showing that the involved bowel segment could be adequately exteriorized through the same incision. Resection was performed through the same incision when adequate exposure and mobilization could be achieved. Laparotomy was performed when the planned procedure could not be safely completed through the limited incision or when adequate intra-abdominal assessment was required.

### 2.4. Data Collection and Endpoints

Demographic characteristics, body mass index (BMI), surgical indication, ASA physical status classification [21], Clinical Frailty Scale (CFS) [22], type of anesthesia administered, surgical procedure, use of intraoperative endoluminal confirmation, and data related to the postoperative clinical course were retrospectively obtained from patient files and the institutional electronic medical record system. The Charlson Comorbidity Index [23] was retrospectively calculated from the available medical records to standardize the assessment of patients’ comorbidity burden.

The primary endpoint of the study was technical feasibility, defined as completion of the planned trephine approach without conversion to laparotomy. The feasibility of performing bowel resection through the same incision was recorded separately as a technical observation and was not considered an efficacy endpoint.

Short-term outcomes included operative time, time to stoma function, time to initiation of oral intake, postoperative complications, length of hospital stay, need for reintervention, 30-day mortality, and 30-day readmission. Stoma-specific complications were recorded separately and evaluated independently of overall postoperative morbidity. The severity of postoperative complications was assessed according to the Clavien–Dindo classification [24]; grade III or higher complications were considered major complications.

### 2.5. Ethical Approval

The study was conducted following approval from the Çukurova University Faculty of Medicine Clinical Research Ethics Committee (Meeting Date: 2 April 2026, Number: 8, Decision No: 5). All data were evaluated retrospectively and in an anonymized form.

### 2.6. Statistical Analysis

Data analysis was performed using IBM SPSS Statistics, version 22.0 (IBM Corp., Armonk, NY, USA). Given the descriptive nature of the study and the limited number of patients, only descriptive statistics were used.

Continuous variables were presented as mean ± standard deviation or median [interquartile range (IQR)] depending on their distribution; categorical variables were presented as number (*n*) and percentage (%).

Since the aim of the study was to describe the feasibility and short-term outcomes of the trephine approach in high-risk geriatric patients, no inferential comparisons or hypothesis tests were performed.

### 2.7. Use of Artificial Intelligence

During the preparation of the manuscript, the authors used ChatGPT (GPT-5; OpenAI, San Francisco, CA, USA) to assist with English-language editing, improving clarity and concision, ensuring terminological consistency, and organizing selected passages in response to peer-review comments. The tool was not used for study design, data collection, statistical analysis, interpretation of the results, or generation of scientific conclusions. All AI-assisted content was critically reviewed and verified by the authors, who take full responsibility for the final content of the manuscript.

## 3. Results

### 3.1. Patient Characteristics

A total of 10 patients who underwent the trephine approach were included in the case series. The mean age was 77.9 ± 9.6 years, and six patients were female. The median body mass index was 24.0 kg/m^2^ (IQR, 18.3–29.5).

The median ASA physical status class was 4 (IQR, 4–4), the median Clinical Frailty Scale (CFS) score was 5.5 (IQR, 4–6), and the median Charlson Comorbidity Index was 5.5 (IQR, 5–7.5). Nine patients were classified as ASA class IV or V. Five patients had a history of previous abdominal surgery, five had a malignancy-related diagnosis, and eight underwent emergency surgery.

Individual patient characteristics are presented in Table 1, and the overall demographic and clinical characteristics of the cohort are summarized in Table 2.

### 3.2. Surgical Indications and Operative Findings

The most common surgical indication was sigmoid volvulus in five patients, followed by rectal cancer in three, an ileal tumor in one, and intestinal obstruction secondary to bladder cancer in one. All patients presented with intestinal obstruction, and eight underwent emergency surgery.

Preoperative CT was performed in all patients for anatomical planning. Intraoperative endoluminal guidance was employed in eight patients when confirmation of bowel orientation was required. The median operative time was 32.5 min (IQR, 30–42.5).

The planned procedure was completed using the trephine approach without conversion to laparotomy in nine patients. One patient required conversion to laparotomy because adequate anatomical orientation could not be achieved through the limited incision. Bowel resection was performed through the same incision in four patients in whom resection was required and considered technically feasible.

Spinal anesthesia was administered in six patients, general anesthesia in three, and local anesthesia in one. Detailed surgical and operative characteristics are summarized in Table 3.

### 3.3. Postoperative Course

The median time to stoma function was 1 day (IQR, 1–2), the median time to oral intake was 2 days (IQR, 2–3), and the median length of hospital stay was 5 days (IQR, 3.8–5.5).

Three patients experienced major postoperative complications (Clavien–Dindo grade ≥ III). One patient with an ileal tumor, who was intubated and in critical condition before emergency surgery, died from multiple organ failure during the early postoperative period (Clavien–Dindo grade V). Two patients developed pleural effusion requiring percutaneous thoracentesis. One of these patients subsequently developed mucocutaneous separation between the stoma and the peristomal skin, requiring early reoperation and surgical re-suturing. The patient later died from multiple organ failure within 30 days (Clavien–Dindo grade V). The other patient recovered without further intervention (Clavien–Dindo grade IIIa).

Mucocutaneous separation requiring surgical re-suturing was the only stoma-specific complication recorded in the series. No other stoma-specific complications were observed. Overall, two patients died within 30 days, one patient required reoperation, and no patient was readmitted within 30 days.

The median follow-up duration was 107.5 days (IQR, 84.5–173.8). None of the seven patients with a loop stoma underwent stoma closure during the available follow-up period, primarily because of persistent surgical risk, overall clinical condition, or the status of the underlying disease. Table 4 summarizes the postoperative outcomes.

## 4. Discussion

This small retrospective case series demonstrates that the trephine approach was technically feasible in selected high-risk older patients requiring stoma surgery, with completion of the planned procedure without conversion to laparotomy in nine of ten patients. This feasibility was observed within a clinical approach that combined assessment of physiological reserve with preoperative CT-based anatomical planning and, when required, intraoperative endoluminal confirmation of bowel orientation. However, three patients experienced major postoperative complications, and two died within 30 days, underscoring that technical feasibility should not be interpreted as evidence of procedural safety or superiority over conventional approaches. Bowel resection through the same incision in four selected patients represents an additional technical observation rather than evidence supporting the trephine approach as a routine resection strategy.

### 4.1. Patient Selection and Physiological Risk

Patient selection is a central consideration when evaluating the trephine approach. In older surgical patients, perioperative risk is influenced not only by chronological age but also by frailty and physiological reserve [4,18]. In our clinical practice, the trephine approach was considered primarily in patients judged to have high surgical risk or limited physiological tolerance of conventional abdominal surgery. This assessment incorporated overall clinical condition, physiological reserve, frailty, comorbidity burden, and anticipated tolerance of a conventional abdominal approach, without predefined ASA or CFS thresholds. The relatively high ASA classifications and CFS values in this series characterize the cohort as a high-risk older population with varying degrees of frailty, rather than a uniformly frail group. The Charlson Comorbidity Index was calculated retrospectively to describe comorbidity burden and was not part of the original clinical decision-making process.

Importantly, the selection process described here was based on individualized multidisciplinary clinical judgment and was neither prospectively defined nor validated as a patient-selection algorithm. Therefore, these findings do not establish specific criteria for selecting patients for trephine stoma. Rather, they provide a preliminary description of a clinical approach that considers physiological risk assessment alongside anatomical suitability when evaluating a less invasive operative option for selected high-risk patients.

### 4.2. Comparison with the Literature

The available literature on trephine stoma consists largely of small and heterogeneous patient series, with considerable variation in indications, patient characteristics, and operative techniques. Nylund et al. reported successful trephine stoma formation in 22 of 27 patients, with conversion required in five patients [13]. Other small series and reviews have also described the trephine approach as a means of creating a stoma without formal laparotomy [9,10]. In a larger series of 263 patients undergoing colostomy formation, the trephine approach was associated with a shorter operative time than open and laparoscopic approaches [25]. A series of 14 patients from Turkey similarly demonstrated the feasibility of trephine loop colostomy under regional anesthesia for heterogeneous indications, including Fournier’s gangrene, inoperable anorectal cancer, and radiotherapy-related stricture [26].

In the present series, the planned procedure was completed without conversion to laparotomy in nine of ten patients. Given the small sample size, retrospective patient selection, and absence of a comparator group, this finding should not be interpreted as indicating a higher technical success rate or superiority relative to previous reports. Instead, it adds descriptive evidence that the trephine approach can be technically completed in carefully selected high-risk older patients, including those presenting predominantly with acute intestinal obstruction.

### 4.3. Imaging and Anatomical Planning

Limited visualization and difficulty in identifying and orienting the intended bowel segment are recognized technical limitations of the trephine approach [11]. In the present series, preoperative CT was used in all patients to assess the level of obstruction, the intended bowel segment, bowel dilatation, the relationship of the target segment to the abdominal wall, abdominal wall anatomy, free intra-abdominal fluid, and the anticipated need for formal intra-abdominal exploration. Anatomical suitability therefore represented an important consideration in addition to physiological risk when the trephine approach was evaluated.

Intraoperative endoluminal guidance provided an additional means of confirming bowel orientation when required. In sigmoid stomas, when the proximal and distal limbs could not be confidently distinguished after exteriorization of the colon, rectoscopy or colonoscopy and/or transanal air insufflation was used to identify the distal segment. Endoscopy-assisted trephine stoma has been described previously [15,16]; however, in our series, endoluminal guidance was not a routine component of every procedure but an adjunct used when anatomical orientation required confirmation. The completion of the planned procedure without laparotomy in nine of ten patients should therefore be interpreted in the context of both preoperative anatomical assessment and the selective use of intraoperative endoluminal confirmation, rather than as a consequence of the trephine incision alone. The single conversion to laparotomy occurred when adequate anatomical orientation could not be achieved through the limited incision, illustrating the importance of conversion when anatomical certainty cannot be established.

Marked bowel dilatation in patients presenting with acute intestinal obstruction may complicate laparoscopic access, trocar placement, pneumoperitoneum, and intra-abdominal visualization [27,28]. In addition, the physiological effects of pneumoperitoneum may be relevant in patients with limited cardiopulmonary reserve [29]. These considerations contributed to the choice of operative approach in this high-risk population. Conversely, formal laparotomy entails a more extensive abdominal incision and greater operative exposure. Within this clinical context, the trephine approach was considered when a limited-access procedure appeared anatomically feasible and the physiological burden of a conventional abdominal operation was considered undesirable. However, because this study did not include open or laparoscopic comparator groups, no conclusions can be drawn regarding the relative morbidity, safety, or superiority of the trephine approach compared with these alternatives.

### 4.4. Extension to Bowel Resection

The trephine approach was originally described as a limited-access technique for stoma formation without routine laparotomy [6]. Previous reports have shown that bowel resection and stoma formation through the same limited incision may also be technically possible in selected patients, particularly in the setting of sigmoid volvulus [8]. In the present series, bowel resection was performed through the same incision in four patients in whom resection was required and the involved bowel segment could be adequately exteriorized.

This finding should be interpreted as a technical observation rather than evidence supporting an extension of the indications for the trephine approach. The ability to perform resection through the same incision depends on favorable anatomy, adequate bowel mobilization and exposure, and the ability to complete the required procedure without compromising anatomical assessment. Accordingly, these cases do not establish the trephine approach as an alternative to conventional abdominal surgery when formal intra-abdominal exploration or more extensive resection is required.

### 4.5. Anesthesia Strategy

The trephine procedures were performed under different anesthetic techniques, with spinal anesthesia used in six patients, general anesthesia in three, and local anesthesia in one. The choice of anesthetic technique was individualized according to the patient’s clinical condition and perioperative anesthetic assessment. None of the procedures initiated under spinal or local anesthesia required conversion to general anesthesia.

Although previous trephine series have also reported the use of regional or local anesthesia [9,10], the present small descriptive series does not allow comparison of outcomes according to anesthetic technique or determination of whether one strategy is preferable to another. The anesthetic distribution in this cohort should therefore be interpreted descriptively rather than as evidence supporting a specific anesthetic strategy.

### 4.6. Complications and Mortality

Major postoperative complications occurred in three of ten patients, and two patients died within 30 days. These outcomes warrant particular caution when interpreting the technical feasibility observed in this series. The complication burden was higher than that reported in some small trephine series involving different and potentially lower-risk patient populations [12,30]. However, direct comparison is inappropriate because of substantial differences in patient selection, operative indications, physiological risk, and the proportion of emergency procedures.

Both 30-day deaths were attributed to multiple organ failure. One occurred in a patient who was already intubated and critically ill before emergency surgery. The other occurred in a patient who developed postoperative pleural effusion and subsequently required reoperation for mucocutaneous separation between the stoma and the peristomal skin. Mucocutaneous separation was the only stoma-specific complication observed in the series. These events emphasize that successful completion of a limited-access procedure does not necessarily translate into a favorable postoperative course in patients with substantial preoperative physiological risk.

Importantly, the absence of a comparator group prevents attribution of postoperative complications or mortality either to the trephine approach itself or to the underlying clinical condition of the patients. Accordingly, the present findings support technical feasibility in selected patients but do not establish procedural safety or demonstrate a reduction in morbidity or mortality compared with conventional abdominal approaches.

### 4.7. Clinical Considerations for Patient Selection

The clinical considerations described in this series were not prospectively defined or validated, and we did not use fixed ASA or CFS thresholds. They should therefore not be interpreted as a patient-selection algorithm or as criteria for recommending trephine stoma formation. Rather, they describe the factors considered in our clinical practice and may serve as a hypothesis-generating framework for future prospective evaluation. Figure 1 summarizes these clinical considerations.

### 4.8. Limitations and Strengths

This study has several important limitations. First, its retrospective, single-center design and small sample size carry a risk of selection bias and limit the generalizability of the findings. Patient selection was based on individualized multidisciplinary clinical judgment rather than prospectively defined criteria or fixed ASA or CFS thresholds. Although frailty assessment tools based on predefined numerical criteria have been described in surgical populations [19], no such algorithm was used to determine eligibility for the trephine approach in the present series. Moreover, some high-risk patients considered for the trephine approach ultimately underwent conventional surgery because of unfavorable anatomy or the anticipated need for formal intra-abdominal exploration; however, these patients were not systematically identifiable in the database. Therefore, the 488 patients who underwent stoma surgery during the study period should not be regarded as a comparator population.

Second, the absence of an open or laparoscopic control group prevents conclusions regarding the relative safety, morbidity, mortality, or clinical benefit of the trephine approach. Although ASA and CFS were routinely assessed preoperatively as part of the clinical evaluation of physiological risk [5], CFS obtained during acute illness may not fully reflect baseline frailty. The Charlson Comorbidity Index was calculated retrospectively for descriptive purposes and was not part of the original clinical decision-making process. Finally, although 30-day outcomes were complete for all patients, longer-term follow-up was less uniform, limiting reliable assessment of late stoma-related complications, quality of life, and stoma reversal.

Nevertheless, this study provides detailed patient-level information on an infrequently reported approach in a highly selected group of high-risk older patients. Preoperative CT-based anatomical planning in all patients and selective intraoperative endoluminal confirmation provide a detailed description of the technical considerations accompanying the trephine approach in this series. In addition, bowel resection through the same incision in four anatomically selected patients represents preliminary technical experience that may warrant further investigation but should not be interpreted as evidence of broader safety or therapeutic efficacy.

## 5. Conclusions

In this retrospective study, the trephine approach was technically feasible in selected high-risk older patients requiring stoma surgery, with completion of the planned procedure without laparotomy in nine of ten patients. Preoperative CT-based anatomical planning and selective intraoperative endoluminal confirmation were important components of the operative approach. Given the small sample size, highly selected population, and absence of a comparator group, these findings should not be interpreted as evidence of procedural safety or superiority over conventional approaches. Larger prospective, multicenter studies are needed to better define patient selection and the clinical role of the trephine approach.

## Figures and Tables

**Table 1:** Patient-Based Summary (Diagnosis, Risk Scores, Resection, and Outcome).

Patient	Diagnosis	ASA	CFS	Charlson	Surgical Setting	Bowel Resection	Conversion	Highest Clavien–Dindo Grade	30-Day Mortality
1	Sigmoid volvulus	IV	6	5	Emergency	No	No	None	No
2	Sigmoid volvulus	IV	7	8	Emergency	Yes	No	None	No
3	Ileal tumor	V	8	8	Emergency	Yes	No	V	Yes
4	Rectal cancer	IV	5	6	Emergency	No	No	None	No
5	Sigmoid volvulus	IV	6	8	Emergency	No	No	V	Yes
6	Rectal cancer	IV	4	5	Elective	No	No	None	No
7	Rectal cancer	IV	4	6	Emergency	No	Yes	IIIa	No
8	Sigmoid volvulus	III	3	4	Emergency	Yes	No	None	No
9	Sigmoid volvulus	IV	3	5	Emergency	Yes	No	None	No
10	Bladder cancer	IV	6	5	Elective	No	No	None	No

ASA, American Society of Anesthesiologists Physical Status Classification; CFS, Clinical Frailty Scale; Charlson, Charlson Comorbidity Index; Conversion, conversion from trephine approach to laparotomy; Highest Clavien–Dindo grade, highest postoperative complication grade recorded within 30 days.

**Table 2:** Patient Demographics and Clinical Characteristics.

Variable	Value
Number of patients	10
Age, years, mean ± SD	77.9 ± 9.6
Female sex, *n* (%)	6 (60.0)
Body mass index, kg/m^2^, median (IQR)	24.0 (18.3–29.5)
ASA physical status, median (IQR)	4 (4–4)
ASA class ≥ 4, *n* (%)	9 (90.0)
Clinical Frailty Scale, median (IQR)	5.5 (4–6)
Charlson Comorbidity Index, median (IQR)	5.5 (5–7.5)
Previous abdominal surgery, *n* (%)	5 (50.0)
Malignant etiology, *n* (%)	5 (50.0)
Emergency surgery, *n* (%)	8 (80.0)

Data are presented as mean ± standard deviation (SD), median (interquartile range [IQR]), or number (%). ASA = American Society of Anesthesiologists Physical Status Classification; IQR = interquartile range.

**Table 3:** Surgical Indications and Operative Characteristics.

Variable	Value
**Surgical indication**	
Sigmoid volvulus	5 (50.0)
Rectal tumor	3 (30.0)
Ileal tumor	1 (10.0)
Bladder tumor	1 (10.0)
**Operative characteristics**	
Emergency surgery	8 (80.0)
Preoperative CT planning	10 (100.0)
Intraoperative endoscopic guidance	8 (80.0)
Operative time, min, median (IQR)	32.5 (30–42.5)
Bowel resection	4 (40.0)
Conversion to laparotomy	1 (10.0)
**Procedure**	
Colostomy	8 (80.0)
Ileostomy	2 (20.0)
Loop stoma	7 (70.0)
Hartmann procedure	3 (30.0)
**Anesthesia**	
Spinal	6 (60.0)
General	3 (30.0)
Local	1 (10.0)

**Table 4:** Postoperative Outcomes.

Variable	Value
Time to stoma function, days, median (IQR)	1 (1–2)
Time to oral intake, days, median (IQR)	2 (2–3)
Length of hospital stay, days, median (IQR)	5 (3.8–5.5)
Major complications (Clavien–Dindo grade ≥ III), *n* (%)	3 (30.0)
Redo surgery, *n* (%)	1 (10.0)
30-day mortality, *n* (%)	2 (20.0)
30-day readmission, *n* (%)	0 (0.0)
Follow-up duration, days, median (IQR)	107.5 (84.5–173.8)

Data are presented as median (interquartile range [IQR]) or number (%). Major complications were defined as Clavien–Dindo grade III or higher.

## Data Availability

The data presented in this study are available on request from the corresponding author due to privacy and ethical restrictions.
